# Higher abundance of *Faecalibacterium prausnitzii* in the gut microbiome is associated with a lower risk of sepsis development among 6,372 individuals followed for 20 years

**DOI:** 10.1128/msystems.00645-26

**Published:** 2026-07-10

**Authors:** Hassan Diab, Robert F. J. Kullberg, Li-Fang Yeo, Irina Wikki, Veikko Salomaa, Aki Havulinna, Leo Lahti, Katariina Pärnänen, Sirpa Jalkanen, Marko Salmi, Max Nieuwdorp, Rob Knight, W. Joost Wiersinga, Joonatan Palmu, Teemu Niiranen

**Affiliations:** 1 Division of Medicine, Turku University Hospital 1041, Turku, Finland; 2 Department of Internal Medicine, University of Turku 8058, Turku, Finland; 3 Center for Infection and Molecular Medicine (CIMM), Amsterdam UMC, University of Amsterdam 1234, Amsterdam, the Netherlands; 4 Institute for Molecular Medicine Finland, FIMM-HiLIFE 168536 , Helsinki, Finland; 5 Department of Computing, University of Turku 8058, Turku, Finland; 6 Department of Microbiology, University of Helsinki 3835, Helsinki, Finland; 7 MediCity Research Laboratory, University of Turku 8058, Turku, Finland; 8 InFLAMES Flagship, University of Turku 8058, Turku, Finland; 9 Institute of Biomedicine, University of Turku 8058, Turku, Finland; 10 Department of Internal Medicine, Division of Vascular Medicine, Amsterdam UMC, University of Amsterdam 1234, Amsterdam, the Netherlands; 11 Department of Pediatrics, University of California San Diego 8784, La Jolla, California, USA; 12 Center for Microbiome Innovation, University of California San Diego 8784, La Jolla, California, USA; 13 Department of Computer Science and Engineering, University of California San Diego 8784, La Jolla, California, USA; 14 Shu Chien-Gene Lay Department of Bioengineering, University of California San Diego 8784, La Jolla, California, USA; 15 Halıcıoğlu Data Science Institute, University of California San Diego 8784, La Jolla, California, USA; 16 Hong Kong University of Science and Technology Jockey Club Institute for Advanced Study, Hong Kong University of Science and Technology, Hong Kong SAR, China; 17 Department of Internal Medicine, Division of Infectious Diseases, Amsterdam UMC, University of Amsterdam 1234, Amsterdam, the Netherlands; 18 Department of Public Health, Finnish Institute for Health and Welfare 3837, Helsinki, Finland; The University of Hong Kong, Hong Kong, Hong Kong

**Keywords:** sepsis, gut microbiome, prospective study, *Faecalibacterium prausnitzii*

## Abstract

The human gut microbiome has been suggested to be linked with the risk of developing sepsis, a life-threatening medical emergency. However, it remains unclear whether the gut microbiome is an independent predictor of long-term sepsis risk in the general adult population. Here, we investigated for the first time the prospective association between the gut microbiome and incident sepsis in the general population. The study sample (FINRISK) consisted of 6,372 individuals who underwent fecal sampling in 2002 and were followed for incident sepsis. We used multivariable-adjusted models to study the associations of microbial alpha-diversity, beta-diversity, taxa, butyrate producers, and predicted pathways with incident sepsis. Two hundred and forty participants developed sepsis over a follow-up of 19.8 years. A 1-SD increase in *Faecalibacterium prausnitzii_C_71351* abundance was associated with 21% (95% CI, 10%–30%; FDR = 0.03) lower risk of sepsis. Higher abundances of six other species were associated with higher sepsis risk (FDR < 0.05 for all). Five of these species were positively associated with C-reactive protein. The species-sepsis associations were consistent across various subgroups. Moreover, in an independent validation cohort of 4,248 individuals, we found a similar association between *Faecalibacterium* and a lower risk of future sepsis. Additionally, overall pathways related to carbohydrate degradation, energy production, and sulfur metabolism were positively linked to incident sepsis. We did not detect any associations of alpha-diversity, beta-diversity, or butyrate producers with incident sepsis. Future studies should investigate the causality of these associations and the mechanisms by which the identified species may influence sepsis development.

**IMPORTANCE:**

Previous cross-sectional and case-control studies have linked changes in the gut microbiome with the occurrence of sepsis. However, the relationship between the gut microbiome and the risk of incident sepsis in the general adult population remains unexplored. Here, we found clear evidence on the association of gut microbiome species with incident sepsis in a large population cohort. In particular, we provided an in-depth analysis of the negative link between *F. prausnitzii* and sepsis risk, which was robust across independent cohorts. This finding supports a potential protective role of *F. prausnitzii*, but further experimental investigation is required. We also show that six species, including *Clostridium symbiosum*—a causative agent of bacteremia/sepsis in few cases—are positively linked to incident sepsis. Most of these species were also positively linked to an inflammatory marker. Our research provides the groundwork for future experimental analysis of the detected associations to understand their role in infection.

## INTRODUCTION

Sepsis is a condition characterized by a dysregulated systemic immune response to infection that can lead to organ dysfunction and potentially death (1). The incidence of hospital-treated adult sepsis cases in high-income countries is estimated at 189 cases per 100,000 person-years (2). Due to the absence of a treatment specific to sepsis (1), it is essential to study different conditions that might contribute to sepsis development. The gut microbiome may be a key factor that impacts sepsis development and outcomes (3). Disruption of the gut microbiome is thought to increase the risk of sepsis through promoting the growth of pathogenic bacteria, influencing the immune response to sepsis, and lowering the secretion of specific microbial metabolites (4). Additionally, increased antimicrobial resistance in the gut microbiome has been linked to a higher risk of sepsis in the general population (5).

Previous studies have reported differences in the gut microbiome composition and diversity between septic and healthy individuals (6–12), highlighting the importance of exploring gut microbiome-sepsis associations. However, these studies have been limited to relatively small sample sizes (*N* = 6–235) and have been mainly cross-sectional or case-control studies (6, 8, 9). These study settings are subject to selection bias, do not make causal inference possible, and do not provide clarity of temporal sequence (i.e., did disruption of the gut microbiome precede the occurrence of sepsis?). Prospective cohort studies, on the other hand, provide a higher level of evidence and causality than cross-sectional and case-control studies. To our knowledge, the relationship between the gut microbiome and the risk of incident sepsis has not yet been investigated in a prospective population-based cohort. The few prospective studies to date have been performed mainly in preterm infants or neonates and have aimed at investigating the relation of gut microbiome evolution with sepsis risk over limited maximum follow-ups of 12 weeks (10–12). One other prospective study has linked gut colonization by gram-negative bacteria to a higher risk of gram-negative bloodstream infections in allogeneic hematopoietic cell transplant patients (13). Additionally, many animal studies have explored the relationship between the gut microbiome and its metabolites with sepsis (14–17). While previous studies have been crucial to our understanding of gut microbiome-sepsis associations, it remains unclear whether the gut microbiome is an independent predictor of long-term sepsis risk in the general adult population.

Here, we examined for the first time the long-term prospective links between the gut microbiome and incident sepsis in 6,372 participants of the FINRISK 2002 prospective population-based cohort. Incident sepsis was observed in 240 participants over a median follow-up period of approximately 20 years. We mainly focused on the associations of gut microbiome diversity (alpha- and beta-diversity), differentially abundant taxa, butyrate producers, and predicted functional pathways with incident sepsis. To account for potential geographic differences in gut microbiota and possible technical influences, we used an independent cohort for validation: 4,248 participants from the Dutch Healthy Life in an Urban Setting (HELIUS) study, with a median follow-up period of approximately 5.5 years.

## RESULTS

A summary of the baseline characteristics of the FINRISK study sample is presented in Table 1. The mean age of the study sample was 49.1 ± 12.8 years, and 46.5% were men. Incident sepsis was observed in 240 participants (3.8%) over a median follow-up of 19.8 years.

**TABLE 1:** Characteristics of the FINRISK study sample^*b*^

Characteristic	All (*N* = 6,372)	Incident sepsis (*N* = 240)	No incident sepsis (*N* = 6,132)	*P*-value^*a*^
Men, *N* (%)	2,965 (46.5%)	141 (58.8%)	2,824 (46.1%)	<0.001
Age (years), mean (SD)	49.1 (12.8)	59.7 (9.80)	48.7 (12.7)	<0.001
BMI (kg/m^2^), mean (SD)	26.9 (4.63)	29.0 (5.34)	26.9 (4.58)	<0.001
Physical activity level, *N* (%)				0.14
Sedentary	1,327 (20.8%)	52 (21.7%)	1,275 (20.8%)	
Light	3,566 (56.0%)	145 (60.4%)	3,421 (55.8%)	
Moderate to high	1,479 (23.2%)	43 (17.9%)	1,436 (23.4%)	
Smoking, *N* (%)	1,526 (23.9%)	60 (25.0%)	1,466 (23.9%)	0.76
Alcohol intake (g/week), mean (SD)	81.7 (123)	88.8 (159)	81.5 (121)	0.50
Diabetes, *N* (%)	266 (4.2%)	31 (12.9%)	235 (3.8%)	<0.001
Cardiovascular disease, *N* (%)	223 (3.5%)	26 (10.8%)	197 (3.2%)	<0.001
Cancer, *N* (%)	179 (2.8%)	17 (7.1%)	162 (2.6%)	<0.001
Liver disease, *N* (%)	44 (0.7%)	6 (2.5%)	38 (0.6%)	0.002
Autoimmune disease, *N* (%)	127 (2%)	14 (5.8%)	113 (1.8%)	<0.001
Beta blocking agent use, *N* (%)	627 (9.8%)	58 (24.2%)	569 (9.3%)	<0.001
Diuretic use, *N* (%)	195 (3.1%)	26 (10.8%)	169 (2.8%)	<0.001
Calcium channel blocker use, *N* (%)	262 (4.1%)	26 (10.8%)	236 (3.8%)	<0.001
RAA system blocker use, *N* (%)	518 (8.1%)	56 (23.3%)	462 (7.5%)	<0.001
Proton pump inhibitor use, *N* (%)	166 (2.6%)	11 (4.6%)	155 (2.5%)	0.08
Antidiabetic drug use, *N* (%)	150 (2.4%)	23 (9.6%)	127 (2.1%)	<0.001
Died within 1 year of follow-up	17 (0.3%)	3 (1.3%)	14 (0.2%)	0.02
Follow-up time (years), median (minimum, maximum)	19.8 (0.03, 20.7)	12.4 (0.23, 20.7)	19.8 (0.03, 20.0)	< 0.001

^*a*^ A *t*-test was used for continuous variables and a χ^2^ test for categorical variables.

^*b*^ SD, standard deviation; BMI, body mass index; RAA, Renin-Angiotensin-Aldosterone.

### Microbiome diversity and composition

No association between gut microbiome alpha diversity (Shannon index) and incident sepsis was observed in the age- and sex-adjusted model (hazard ratio [HR] per 1-standard deviation [SD] change 0.96; 95% confidence interval [CI] 0.85–1.09; *P*  =  0.53) or in the multivariable-adjusted model (HR per 1-SD change 1; CI 0.88–1.14; *P*  =  0.98,) which was adjusted for age, sex, BMI, smoking, alcohol use, physical activity, prevalent diabetes, prevalent cardiovascular disease, and previous cancer diagnosis. Similarly, gut microbiome beta-diversity was not associated with incident sepsis in the age- and sex-adjusted (R^2^ = 0.02%; *P* = 0.06) or multivariable-adjusted (R^2^ = 0.02%; *P* = 0.12) supervised ordination distance-based redundancy (dbRDA) models. Using Random Survival Forests, we observed that the assessment of incident sepsis risk was not improved by the addition of prevalent taxa to the model. The *c*-statistic for covariates alone (0.754) was higher than the *c*-statistic for covariates plus prevalent taxa (0.730). The *c*-statistic for prevalent taxa alone was 0.591. The top 20 species with the highest importance scores (Table S1) are presented in Fig. S1.

### Differential abundance analysis

**Fig 1:**
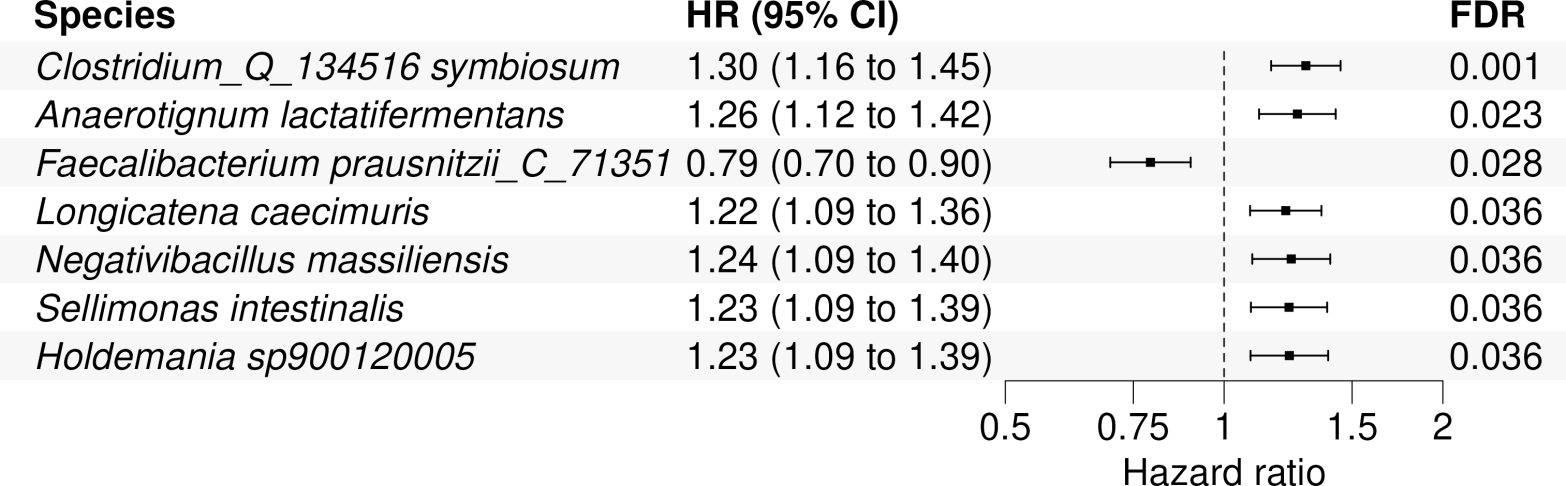
Species significantly associated with incident sepsis. Hazard ratios are reported per one standard deviation change in CLR-transformed abundance and are adjusted for age, sex, BMI, smoking, alcohol use, physical activity, prevalent diabetes, prevalent cardiovascular disease, and previous cancer diagnosis. HR, hazard ratio; CI, confidence interval; FDR, false discovery rate-adjusted *P*-value.

**Fig 2:**
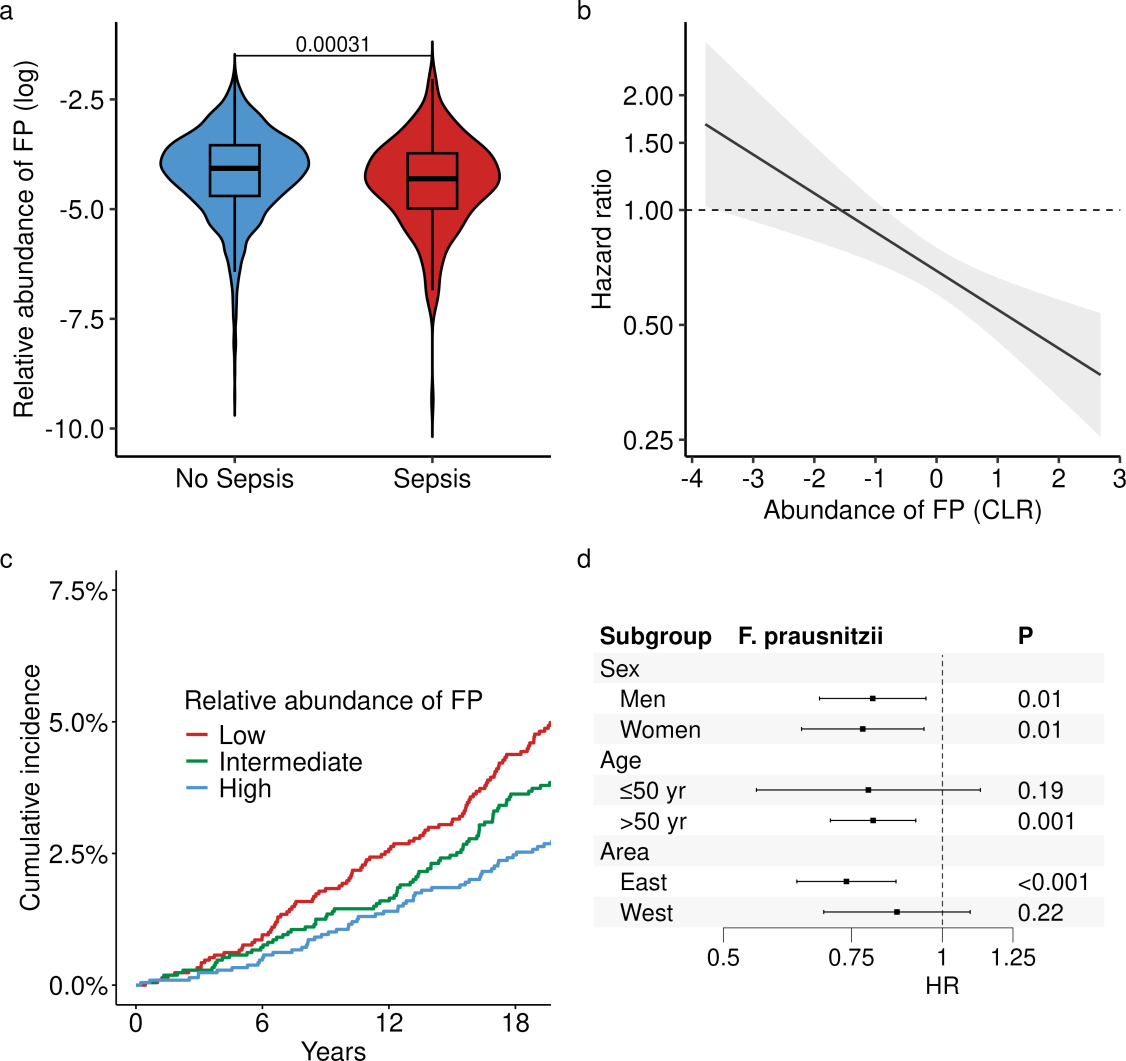
*F. prausnitzii* is associated with a lower risk of incident sepsis. (**a**) The relative abundance of *F. prausnitzii* (log-transformed) in participants with and without sepsis. The *P*-value of the Wilcoxon test is indicated. (**b**) Predicted hazard ratio values based on *F. prausnitzii* abundances (CLR-transformed and scaled) and obtained using a multivariable-adjusted Cox model. (**c**) Cumulative incidence of sepsis stratified by tertiles of *F. prausnitzii* relative abundance. (**d**) Subgroup analysis of *F. prausnitzii*. The association of *F. prausnitzii* with incident sepsis was tested in each subgroup using multivariable-adjusted Cox models. Hazard ratios are reported per one standard deviation change in CLR-transformed abundance and are adjusted for age, sex, BMI, smoking, alcohol use, physical activity, prevalent diabetes, prevalent cardiovascular disease, and previous cancer diagnosis. Tertiles of *F. prausnitzii* were defined based on relative abundance and included low (relative abundance ≤ 0.0115230), intermediate (0.0115230 < relative abundance ≤ 0.0239029), and high (relative abundance > 0.0239029). FP, *F. prausnitzii*; HR, hazard ratio; P, *P*-value.

**Fig 3:**
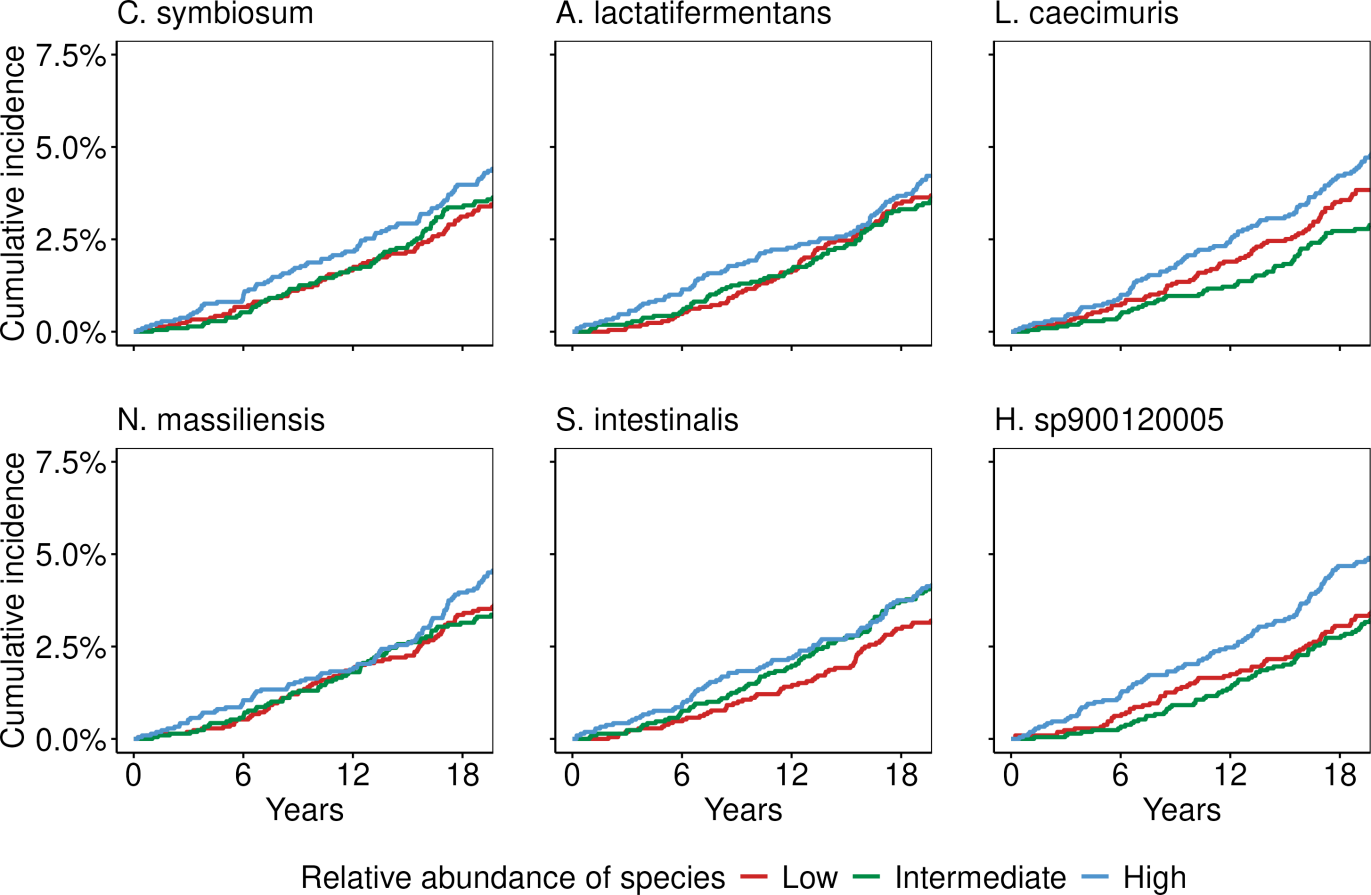
Cumulative incidence of sepsis stratified by tertiles of relative abundances for species associated with a higher risk of sepsis.

Cox regression analysis with multivariable-adjusted models revealed that a 1-SD increase in the centered log-ratio (CLR)-transformed abundance of *Faecalibacterium prausnitzii_C_71351* was associated with a 0.79-fold (95% CI, 0.70–0.90; FDR = 0.03) risk of incident sepsis, whereas *Clostridium_Q_134516 symbiosum*, *Anaerotignum lactatifermentans*, *Longicatena caecimuris*, *Negativibacillus massiliensis*, *Sellimonas intestinalis*, and *Holdemania sp900120005* were positively associated with a higher risk of incident sepsis (FDR < 0.05 for all) (Fig. 1 and Fig. 2c and Fig. 3 and Table S2). Consistent results were observed at the genus level with two additional associations detected for *Bacteroides_F* and *Butyricicoccus_A_77030* (Table S3). The species that were positively associated with incident sepsis were positively correlated (Fig. S2). The CLR-transformed abundance of butyrate-producing bacteria was not associated with incident sepsis in the age- and sex-adjusted (HR per 1-SD change 0.97; CI 0.85–1.10; *P*  =  0.60) or multivariable-adjusted (HR per 1-SD change 0.95; CI 0.84–1.07; *P* = 0.41) models.

In in-depth analyses on *F. prausnitzii_C_71351*, the relative abundance of this species was significantly lower in individuals who developed sepsis (mean = 0.0190 ± .020) compared to those who did not develop sepsis (mean = 0.0222 ± .019) (Wilcoxon *P* = 0.0003) (Fig. 2A). The association between CLR-transformed *F. prausnitzii_C_71351* abundance and incident sepsis was linear (Fig. 2B). *F. prausnitzii_C_71351* was generally not correlated with the rest of the significant species (Fig. S2). The distribution of *F. prausnitzii_C_71351* relative abundance across participants is shown in Fig. S3. A summary of participants’ characteristics across tertiles of *F. prausnitzii_C_71351* abundance is presented in Table S4. The Spearman correlation coefficients of *F. prausnitzii_C_71351* abundance with age, BMI, and alcohol consumption were −0.06, 0.009, and 0.03, respectively.

### CRP and inflammatory cytokines

Linear regression with multivariable-adjusted models revealed a positive association of inflammatory marker C-reactive protein (CRP) with *S. intestinalis* (β = 0.09 ± .02/SD, *P* = 0.00001), *H. sp900120005* (β = 0.06 ± .02/SD, *P* = 0.001), *A. lactatifermentans* (β = 0.06 ± .02/SD, *P* = 0.001), *L. caecimuris* (β = 0.05 ± .02/SD, *P* = 0.01), and *C. symbiosum* (β = 0.05 ± .02/SD, *P* = 0.02) (Fig. S4 and Table S5). Inflammatory cytokines that were positively associated with the tested species (species positively correlated with sepsis) were interleukin (IL)−8, IL-6, interferon-γ (IFN-γ), and macrophage inflammatory protein-1β (MIP-1β) (Fig. S4 and Table S5).

### Sensitivity analyses

**Fig 4:**
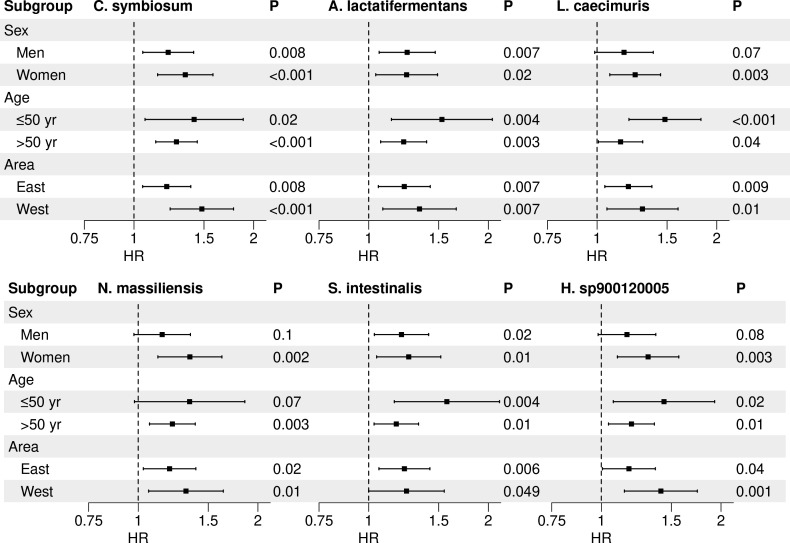
Subgroup analysis of the species associated with a higher risk of incident sepsis. The association of taxa with incident sepsis was tested in each subgroup using multivariable-adjusted Cox models. Hazard ratios are reported per one standard deviation change in CLR-transformed abundance and are adjusted for age, sex, BMI, smoking, alcohol use, physical activity, prevalent diabetes, prevalent cardiovascular disease, and previous cancer diagnosis. Subgroups of men (*N* = 2,965, incident cases = 141), women (*N* = 3,407, incident cases = 99), ≤50 years old (*N* = 3,157, incident cases = 31), >50 years old (*N* = 3,215, incident cases = 209), east (*N* = 4,348, incident cases = 165), and west (*N* = 2,024, incident cases = 75) were used. HR, hazard ratio; P, *P*-value; yr, year.

The hazard ratios of the significant species remained consistent across subgroups of sex, age, and geographical area (Fig. 2d and 4 and Table S6). The significant species that were discovered in the full cohort were found significantly associated with incident sepsis in all subgroups with the following exceptions: *F. prausnitzii_C_71351* in ≤50-year-old individuals (*P* = 0.2) and western Finland (*P* = 0.22), *L. caecimuris* in men (*P* = 0.07), *N. massiliensis* in men (*P* = 0.1) and ≤50-year-old individuals (*P* = 0.07), and *H. sp900120005* in men (*P* = 0.08). Additionally, the identified associations remained significant (with consistent hazard ratios) when the analysis was performed for only 10 years of follow-up (Table S7).

### Functional analysis

No significant associations were detected between predicted species-stratified metabolic pathways and incident sepsis using multivariable-adjusted Cox models with inverse-rank normalized count data (Table S8) or dichotomized count data (present vs. absent) (Table S9). When overall pathways (inverse-rank normalized count data) were used, we observed a positive association between incident sepsis and six pathways related to carbohydrate degradation (glucose, starch, and trehalose), energy production, and sulfur compound metabolism (Table S10). No significant associations were detected between overall pathways and incident sepsis with dichotomized count data (Table S11).

### Independent validation

Because *F. prausnitzii* was the only taxon associated with a lower risk of incident sepsis, we assessed whether *Faecalibacterium* showed a similar association in the independent validation cohort. Demographic and clinical characteristics of the 4,248 included participants have been previously published (18). Incident sepsis was observed in 29 participants (0.68%) over a median follow-up of 5.55 years. In the validation cohort, the relative abundance of *Faecalibacterium* was 7.24% (IQR: 3.95%–9.21%) in individuals who developed sepsis compared to 8.35% (IQR 5.75%–11.34%) in those who did not develop sepsis, which was borderline significant (Wilcoxon *P* = 0.054) (Fig. S5). In a univariable Cox model, the hazard ratio for the association between continuous *Faecalibacterium* abundance and incident sepsis was similar to that observed in the derivation cohort (HR 0.75, 95% CI 0.51—1.10, *P* = 0.145; Fig. S5), although the estimate did not reach statistical significance.

## DISCUSSION

In this study, we explored for the first time the role of gut microbiome as a risk factor for incident sepsis in a population-based cohort. Over a median follow-up period of approximately 20 years, 240 participants (out of 6,372 participants) developed sepsis. We observed that greater *F. prausnitzii* abundance was associated with a lower risk of incident sepsis, whereas greater abundances of *C. symbiosum*, *A. lactatifermentans*, *L. caecimuris*, *N. massiliensis*, *S. intestinalis*, and *H. sp900120005* were associated with a higher risk of incident sepsis. Consistent results were observed across subgroups of sex, age, and geographic area and when follow-up time was limited to 10 years only. Of note, only three participants (1.3%) who developed sepsis died within the first year of follow-up, which therefore had no relevant effect on the results. The finding that the relative abundance of *F. prausnitzii* was lower in individuals who developed sepsis compared with those who did not develop sepsis was robust across cohorts, despite differences in geographical locations and microbiota characterization methods. Moreover, we observed a positive link between incident sepsis and overall pathways related mainly to carbohydrate degradation, energy production, and sulfur metabolism. We did not detect any associations between microbial alpha-diversity, beta-diversity, or butyrate producers with incident sepsis.

We observed that a higher abundance of *F. prausnitzii* in the gut microbiome was associated with a lower risk of incident sepsis, suggesting a potential protective role of *F. prausnitzii* against sepsis. Several case-control studies have reported a lower abundance of *F. prausnitzii* or genus *Faecalibacterium* in sepsis patients compared with healthy controls (6, 8, 19). These studies compared the composition of the gut microbiome between individuals with or without sepsis and hence did not provide information on the direction of association between *F. prausnitzii* or genus *Faecalibacterium* with sepsis risk. Prospective studies, on the other hand, mainly focused on changes in the gut microbiome composition prior to the development of sepsis and have been performed mainly in preterm infants or neonates (10–12). Our study provided strong evidence on the negative link between higher abundance of *F. prausnitzii* and the risk of developing sepsis in the general adult population.

Several studies have established a protective role of butyrate against bacterial infections, including *C. difficile* infection and bacterial pneumonia (18, 20, 21). In our study, however, the overall abundance of butyrate-producing bacteria was not associated with incident sepsis. Therefore, and based on our analysis, it appears that the potential protective effects of *F. prausnitzii* against sepsis might be independent of its butyrate-producing function. However, it is noteworthy that in our study, we did not measure the amounts of fecal butyrate in participants. The link between butyrate production by the gut microbiome and incident sepsis may be more accurately studied using measured levels of fecal butyrate.

Another important finding in this study was the association of *C. symbiosum* and genus *Clostridium* with a higher risk of incident sepsis. Despite being a rare condition, clostridial bacteremia is associated with a high mortality rate (22). The gastrointestinal tract has been previously identified as the primary source of *Clostridium* species in clostridial bacteremia (23). *C. symbiosum* and other members of the genus *Clostridium,* such as *C. perfringens* and *C. difficile*, have been previously reported as the causative agents of bacteremia/sepsis in patients (22, 24–29). *C. symbiosum* is also positively linked to various diseases, including sarcopenia and type 2 diabetes, and has been proposed as a potential biomarker for the early diagnosis of colorectal cancer (30–32). Our study, which shows a positive association between *C. symbiosum* and incident sepsis, and previous work, which indicates a similar relationship with other diseases, highlights the importance of this gut microbiome species and its potential role in disease development.

In addition to *C. symbiosum*, five other species, including *A. lactatifermentans*, *L. caecimuris*, *N. massiliensis*, *S. intestinalis*, and *H. sp900120005*, were associated with an increased risk of incident sepsis. *A. lactatifermentans* was among the top taxa with the highest importance scores based on Random Survival Forest analysis. Moreover, these six species (including *C. symbiosum*) were positively correlated with each other but not with *F. prausnitzii*. Additionally, most of these species were positively associated with an inflammatory marker CRP. Little is known about these species and their link to human health. One study reported a higher abundance of *S. intestinalis* in schizophrenia patients compared to two control groups (33). Another study involving chronic kidney disease patients observed an increase in the abundance of *N. massiliensis* in hemodialysis patients compared to healthy controls (34). The association of these species with infection is not yet understood, and therefore, more research efforts are required to confirm their role in sepsis and human health.

The strengths of our study include a randomly selected large population sample and the use of shotgun metagenome sequencing. In addition, we validated the association between *Faecalibacterium* and incident sepsis in an independent validation cohort to account for potential differences in participants’ characteristics and technical factors. To our knowledge, this was the first study to examine the association between the gut microbiome and incident sepsis prospectively over the long term. Our study, however, is limited by several factors. First, the FINRISK cohort is composed of participants with Finnish or European ancestry, which prevents the generalization of all results to other populations. However, the main finding of this study was validated in a multiethnic population-based cohort. Second, the FINRISK cohort is subject to non-participation bias. Third, although our study provides information regarding the direction of the association between specific gut microbiome species and the risk of incident sepsis, causal inference cannot be made, given the observational study design. Fourth, metagenomic sequencing was performed at one time point, and hence, we were unable to record changes in the gut microbiome during the follow-up period. However, previous studies have demonstrated that the gut microbiome in individuals remains stable over the years (35, 36).

### Conclusion

Sepsis remains a major public health challenge, and hence, it is crucial to understand the relationship between this condition and different factors, including the gut microbiome (3). In this study, we assessed for the first time the prospective, long-term association between the gut microbiome and incident sepsis in a large population-based cohort. The main finding was that a 1-SD increase in *F. prausnitzii_C_71351* abundance was associated with a 21% lower risk of sepsis. Such a finding suggests a potential protective role of *F. prausnitzii* against sepsis, which requires further experimental analysis. Another major finding was the positive association between *C. symbiosum* and incident sepsis. *C. symbiosum* was previously reported as the causative agent of sepsis in a few case reports and was also positively associated with different diseases in large-scale studies. Future work should focus on understanding the potential mechanisms through which the detected significant species could possibly confer their effect on the development of sepsis.

## MATERIALS AND METHODS

### Study sample

The FINRISK 2002 cohort is comprised of 25- to 74-year-old individuals randomly selected from six different geographical areas of Finland (37). A sample of 13,437 individuals was randomly drawn from the national population register in 2002. The number of individuals who participated in the health examination was 8,799. Fecal samples were provided by 7,054 participants for which microbiome sequencing was successfully performed. From these participants, we excluded those with antibiotic use within 1 month before baseline (*n* = 242), self-reported pregnancy (*n* = 40), a metagenomics read count <50,000 reads (*n* = 13), and missing covariate data (*n* = 387) for a final study sample of 6,372 individuals who were included in the analyses.

### Questionnaire and health examination

Information about participants’ family history, diagnoses, use of medications, diet, functional capacity, and other health behaviors was collected through a detailed questionnaire. Anthropometric measurements and blood sample collection were performed as part of the health examination at local study centers. Instructions on how to collect a stool sample at home were given to participants at the end of the health examination.

### Microbiome sequencing from stool samples

Stool sample collection was performed at home, and samples were mailed to the Finnish Institute for Health and Welfare overnight. The samples were then stored at −20°C until they underwent metagenomic sequencing in 2017 at the University of California, San Diego. The microbiome analysis was performed by mapping the untargeted shallow shotgun metagenomic reads against reference databases, as previously described (38). Taxonomy was assigned using the Greengenes2 database (39), and the data were imported into an R TreeSummarizedExperiment container (40) for downstream analysis. MetaCyc (41) database in the HUMAnN3 pipeline (42) was used to annotate functional pathway abundances.

### Outcome variable

Incident sepsis was used as an outcome variable. The International Classification of Diseases (ICD) codes used to define sepsis are available in Table S12. The term “incident sepsis” refers to the development of sepsis in participants during the follow-up period.

### Covariates

Height and weight for calculating body mass index (BMI, kg/m^2^) were measured by centrally trained nurses. Smoking was defined as self-reported current daily smoking. Alcohol consumption was self-reported and calculated as the average weekly pure alcohol use in grams during the past 12 months. For physical activity, participants responded to a four‐option multiple choice question that included the following options for leisure‐time activity: (i) sedentary, (ii) light activity for >4 h per week, (iii) fitness training or other strenuous exercise for >3 h per week, and (iv) competitive sports (18). Categories three and four were combined due to the small number of individuals in category four. The ICD codes used to define diabetes, cardiovascular disease, cancer, and other diseases are available in Table S12.

### Independent validation

Data from the large-scale, population-based HELIUS study were used for external validation of the association between *Faecalibacterium* and incident sepsis, as it was the only taxon associated with a reduced risk. Details on study design, microbiome sequencing, and assessment of outcomes have been previously published (18). In summary, HELIUS is a prospective, multiethnic, population-based cohort study (Amsterdam, the Netherlands) in which adults were randomly sampled from the municipality register, stratified by ethnicity, and invited to participate. Participants provided collected stool samples at inclusion (January 2013 through November 2015). Gut microbiota were profiled by 16S rRNA gene sequencing (V4 region) and subsequently preprocessed in Deblur (version 1.1.1). Similar to the FINRISK cohort, taxonomy was assigned using the Greengenes2 database (39). Given the limited resolution of 16S rRNA gene sequencing at the species level, the relationship of *Faecalibacterium* (at the genus level) with outcomes was assessed. Outcome data were retrieved from national registries using non-public microdata (Statistics Netherlands). We used national registry data for events (hospitalization and mortality) that took place after sample collection and between January 1, 2013, and December 31, 2020. Because not all hospitals provided data in 2011 and 2012, the inclusion of participants who submitted a stool sample during this period could introduce immortal time bias (i.e., no events could occur in 2011–2012). Therefore, we excluded all HELIUS participants enrolled before January 1, 2013 (*n* = 1,156), consistent with our previous work (18).

### Statistical methods

We calculated alpha diversity (Shannon index) at species level using the mia R/Bioconductor package (43). For beta-diversity, we used supervised ordination distance-based redundancy analysis (dbRDA) with the Bray-Curtis index to calculate the dissimilarity matrix at the species level. For this analysis, the microbial taxa relative abundance table was used as an outcome (dependent) variable, while incident sepsis was used as a predictor (independent variable). Taxa-level differential abundance analysis was performed for prevalent taxa using multivariable-adjusted Cox proportional hazard models. Prevalent taxa were defined as being prevalent in at least 5% of the sample population with a relative microbial abundance over 0.1%. The prevalent taxa included 173 genera and 259 species. CLR transformation was applied to the abundances of the prevalent taxa. The correlation between the significant species was determined using Spearman rank correlation coefficient. To test for the association of butyrate producers with incident sepsis, we combined the counts of the bacteria that were previously described as the most abundant butyrate producers (listed in Table S13) (18, 44, 45). CLR transformation was then applied to cumulative counts of butyrate producers. Major butyrate-producing taxa were selected based on a study that examined butyrate-production pathways across 2,387 metagenomic and transcriptomic samples from 15 publicly available data sets (44). The accuracy of this method was previously verified in two independent cohorts by examining the correlation between the abundance of butyrate producers and fecal levels of butyrate (46, 47). In particular, the relative abundances of butyrate-producers showed a strong positive correlation with the absolute fecal butyrate concentrations in the same sample (Pearson r = 0.76) (46).

In both the FINRISK (derivation) and HELIUS (validation) cohort, we assessed the difference in the relative abundances of *F. prausnitzii* between participants with and without incident sepsis using the Wilcoxon rank-sum test. We chose the Wilcoxon test with relative abundances because it has shown good performance in recent benchmarks of microbial differential abundance analysis (48, 49).

For functional analyses, we used predicted MetaCyc pathways (41) (both overall and species-stratified) that were prevalent in at least 5% in the sample population. The pathway counts were (i) dichotomized or (ii) inverse-rank normalized to account for the highly sparse and zero-enriched nature of the pathway data. For the dichotomous transformation, counts that were greater than zero were transformed to one. Total read count was added as a covariate to account for its potential confounding role in dichotomous data.

The associations between incident sepsis and (i) alpha diversity, (ii) prevalent taxa (CLR-transformed counts), (iii) butyrate producers (CLR-transformed counts), and (iv) prevalent predicted pathways (dichotomized or inverse-rank normalized counts) were assessed using multivariable-adjusted Cox proportional hazard models. The cumulative incidence of sepsis was estimated and stratified into equal tertiles of relative abundances of the significant taxa (low, intermediate, and high) and visualized using Kaplan-Meier curves. To evaluate the robustness of the results, we used multivariable-adjusted Cox models to assess the associations of the significant taxa with incident sepsis (i) across different subgroups (men vs. women, ≤50 vs. >50-year-old individuals, eastern vs. western Finland) and (ii) for only 10 years of follow-up. In the validation cohort (HELIUS), a univariable Cox proportional hazard model was used. We also checked for the validity of the proportional hazard assumption and observed that Schoenfeld residuals were randomly disturbed over the follow-up time.

We tested whether the species positively associated with incident sepsis were correlated with 16 inflammatory cytokines and CRP in a subset of the FINRISK cohort (participants aged over 50 years; *N* = 2,281). We used multivariable-adjusted linear models to evaluate these associations. The tested 16 inflammatory cytokines, along with proportions of participants (%) with detectable, above or below detection limits, and unmeasured cytokine and CRP values are listed in Table S14. The methods used to measure cytokine and CRP concentrations, as well as the procedures for estimating values above or below detection limits, are described in detail elsewhere (50, 51).

We used multivariate Random Survival Forest (52) (R package randomForestSRC) to investigate the relationship between microbial community composition and incident sepsis, as previously described (38). CLR-transformed counts of the prevalent taxa (species level) were used for this analysis, and Harrell’s *c*-statistic (53) was used to evaluate the performance of the model. The predictor sets included microbiome (prevalent taxa), covariates, and microbiome plus covariates. Prediction errors were estimated using out-of-bag estimators.

Differences in baseline characteristics between the sepsis and no sepsis groups were assessed using *t*-tests for continuous variables and χ^2^ tests for categorical variables. To evaluate whether participants were in an end-of-life situation, we created a new binary variable called “Died within one year of follow-up.” Participants were classified as “Yes” if death occurred within 1 year of the follow-up period and as “No” otherwise.

All models in the derivation cohort (FINRISK) were adjusted for variables known to be associated with sepsis and were age, sex, BMI, smoking, alcohol use, physical activity, prevalent diabetes, prevalent cardiovascular disease, and previous cancer diagnosis. Analyses for alpha-diversity, beta-diversity, and butyrate producers were also performed with age- and sex-adjusted models. *P*-values were corrected for multiple testing using false discovery rate (FDR; Benjamini-Hochberg correction). FDR values < 0.05 were considered statistically significant. R version 4.5.0 was used for all statistical analyses.

## Data Availability

The FINRISK data are available through submitting a request to THL biobank (https://thl.fi/en/our-services/thl-biobank1/how-to-apply-for-thl-biobank-data-and-samples). Data from the HELIUS study can be requested by submitting a proposal to the Amsterdam UMC (http://www.heliusstudy.nl/en/researchers/collaboration), which will be checked for compatibility with the general objectives, ethical approval, and informed consent forms of the HELIUS study. A STORMS (Strengthening The Organizing and Reporting of Microbiome Studies) checklist (54) is available at https://zenodo.org/records/21033868. The underlying code for this study is publicly available and can be accessed via https://zenodo.org/records/17097459.

## SUPPLEMENTAL MATERIAL

Supplemental Figures msystems.00645-26-s0001.docx Fig. S1 to S5.

Supplemental Tables msystems.00645-26-s0002.xlsx Tables S1 to S14.

ASM does not own the copyrights to Supplemental Material that may be linked to, or accessed through, an article. The authors have granted ASM a non-exclusive, world-wide license to publish the Supplemental Material files. Please contact the corresponding author directly for reuse.
