# Supplementary material for: Higher abundance of Faecalibacterium prausnitzii in the gut microbiome is associated with a lower risk of sepsis development among 6,372 individuals followed for 20 years
Source: mSystems. 2026 Jul 10;11(8):e00645-26. doi: 10.1128/msystems.00645-26 (PMC13483378; doi:10.1128/msystems.00645-26)
Supplement: Supplementary file 1 [file msystems.00645-26-s0001.docx]

**SUPPLEMENTARY MATERIAL**

Higher abundance of *Faecalibacterium prausnitzii* in the gut microbiome is associated with a lower risk of sepsis development among 6,372 individuals followed for 20 years

Hassan Diab,^a,b^# Robert F.J. Kullberg,^c^ Li-Fang Yeo,^a,b^ Irina Wikki,^a,b^ Veikko Salomaa,^b^ Aki Havulinna,^d,e^ Leo Lahti,^e^ Katariina Pärnänen,^e,f^ Sirpa Jalkanen,^g,h^ Marko Salmi,^g,h,i^ Max Nieuwdorp,^j^ Rob Knight,^k,l,m,n,o,p^ W. Joost Wiersinga,^c,q^ Joonatan Palmu,^a,b^ Teemu Niiranen^a,b,r^

^a^Division of Medicine, Turku University Hospital, Turku, Finland.

^b^Department of Internal Medicine, University of Turku, Turku, Finland.

^c^Center for Infection and Molecular Medicine (CIMM), Amsterdam UMC, University of Amsterdam, Amsterdam, The Netherlands.

^d^Institute for Molecular Medicine Finland, FIMM-HiLIFE, Helsinki, Finland.

^e^Department of Computing, University of Turku, Turku, Finland.

^f^Department of Microbiology, University of Helsinki, Helsinki, Finland.

^g^MediCity Research Laboratory, University of Turku, Turku, Finland.

^h^InFLAMES Flagship, University of Turku, Turku, Finland.

^i^Institute of Biomedicine, University of Turku, Turku, Finland.

^j^Department of Internal Medicine, Division of Vascular Medicine, Amsterdam UMC, University of Amsterdam, Amsterdam, The Netherlands.

^k^Department of Pediatrics, University of California San Diego, La Jolla, CA, USA.

^l^Center for Microbiome Innovation, University of California San Diego, La Jolla, CA, USA.

^m^Department of Computer Science and Engineering, University of California San Diego, La Jolla, CA, USA.

^n^Shu Chien-Gene Lay Department of Bioengineering, University of California San Diego, La Jolla, CA, USA.

^o^Halıcıoğlu Data Science Institute, University of California San Diego, La Jolla, CA, USA.

^p^Hong Kong University of Science and Technology Jockey Club Institute for Advanced Study, Hong Kong University of Science and Technology, Hong Kong SAR, China.

^q^Department of Internal Medicine, Division of Infectious Diseases, Amsterdam UMC, University of Amsterdam, Amsterdam, The Netherlands.

^r^Department of Public Health, Finnish Institute for Health and Welfare, Helsinki, Finland.

#Address correspondence to Hassan Diab, [hassan.h.diab@utu.fi](mailto:hassan.h.diab@utu.fi)

**Fig. S1** Top 20 species with highest importance scores based on multivariate Random Survival Forest analysis. Random Survival Forest analysis was performed using CLR-transformed counts of the prevalent taxa (species-level) and was adjusted for age, sex, BMI, smoking, alcohol use, physical activity, prevalent diabetes, prevalent cardiovascular disease, and previous cancer diagnosis.

**Fig. S2** Correlation of species that were significantly associated with incident sepsis calculated using Spearman rank correlation coefficient.

**Fig. S3** The distribution of *F. prausnitzii_C_71351* relative abundance across participants. The distribution curve is colored according to *F. prausnitzii* relative abundance tertiles. The tertiles of *F. prausnitzii* were defined based on relative abundance and included low (relative abundance ≤ 0.0115230), intermediate (0.0115230 < relative abundance ≤ 0.0239029), and high (relative abundance > 0.0239029).

 **Fig. S4** Associations of sepsis-associated species with inflammatory cytokines and CRP, as revealed by linear regression. Estimates are reported per 1-SD increment and are adjusted for age, sex, BMI, smoking, alcohol use, physical activity, prevalent diabetes, prevalent cardiovascular disease, and previous cancer diagnosis.

**Fig. S5** Independent validation of the association between *Faecalibacterium* and a lower risk of incident sepsis among 4,248 participants from the HELIUS study. a) The relative abundance of *Faecalibacterium* in participants with and without sepsis. The P-value of the Wilcoxon test is indicated. b) Cumulative incidence of sepsis stratified by tertiles of *Faecalibacterium* relative abundance.
